# Draft whole genome sequence of groundnut stem rot fungus *Athelia rolfsii* revealing genetic architect of its pathogenicity and virulence

**DOI:** 10.1038/s41598-017-05478-8

**Published:** 2017-07-13

**Authors:** M. A. Iquebal, Rukam S. Tomar, M. V. Parakhia, Deepak Singla, Sarika Jaiswal, V. M. Rathod, S. M. Padhiyar, Neeraj Kumar, Anil Rai, Dinesh Kumar

**Affiliations:** 1 Centre for Agricultural Bioinformatics, ICAR-Indian Agricultural Statistics Research Institute, Library Avenue, Pusa, New Delhi 110012 India; 2 Junagadh Agricultural University, Junagadh, 362 001 Gujarat India

## Abstract

Groundnut (*Arachis hypogaea* L.) is an important oil seed crop having major biotic constraint in production due to stem rot disease caused by fungus, *Athelia rolfsii* causing 25–80% loss in productivity. As chemical and biological combating strategies of this fungus are not very effective, thus genome sequencing can reveal virulence and pathogenicity related genes for better understanding of the host-parasite interaction. We report draft assembly of *Athelia rolfsii* genome of ~73 Mb having 8919 contigs. Annotation analysis revealed 16830 genes which are involved in fungicide resistance, virulence and pathogenicity along with putative effector and lethal genes. Secretome analysis revealed CAZY genes representing 1085 enzymatic genes, glycoside hydrolases, carbohydrate esterases, carbohydrate-binding modules, auxillary activities, glycosyl transferases and polysaccharide lyases. Repeat analysis revealed 11171 SSRs, LTR, GYPSY and COPIA elements. Comparative analysis with other existing ascomycotina genome predicted conserved domain family of WD40, CYP450, Pkinase and ABC transporter revealing insight of evolution of pathogenicity and virulence. This study would help in understanding pathogenicity and virulence at molecular level and development of new combating strategies. Such approach is imperative in endeavour of genome based solution in stem rot disease management leading to better productivity of groundnut crop in tropical region of world.

## Introduction

Groundnut (*Arachis hypogaea* L.) is an important oil seed crop, which like other legumes, harbor symbiotic nitrogen-fixing bacteria in root nodules^1, 2^ improving soil fertility. It has global annual production of 43.92 million tonnes in 2014. India is the second largest producer of groundnut with 6.56 million tonnes (FAO, 2014) after China (16.55 million tonnes). One of the major biotic constraints in production is stem rot/ *Sclerotium* wilt disease caused by *Sclerotium (Athelia) rolfsii*. *Athelia rolfsii* is the sexual state of the fungus, but it is rarely seen, in contrast to the asexual state, known as *Sclerotium rolfsii*. It causes average loss of 25%^3^ which goes upto 80% in severe cases^4^. This soil borne pathogen has vast range of host (>500 plant species) due to its ability of high growth rate along with production of cell degrading enzymes^5^.

The fungal mycelium covering plant stem near soil produces organic acid which is toxic to plants^6^. With the help of fungal secretory system, mycelia invade in stem causing rotting of the tissue due to toxicity that further leads to necrosis^7^. The characteristic symptom of the disease is due to abundance of white mycelium along with small brown spherical sclerotia^8^. Biocontrol of *A. rolfsii* has been reported by *Trichoderma* fungi, and chemical control by fungicides such as tebuconazole, pentachloronitrobenzene (PCNB) and flutolanil^9^. Biocontrol has compromised efficacy and chemical control by fungicide has issues of bio-magnification in food chain and associated environmental problems^10^.

Genome sequencing can reveal role of each individual gene and their networks responsible for plant pathogen interaction, growth, evolutionary relationship and genes for pathogenicity. Whole genome sequencing of *Athelia rolfsii* is imperative not only to study the host-pathogen (HP) interaction but such knowledge discovery may lead to more effective disease combating strategy. Annotated genes/ predicted proteins can be directly used as new targets in fungicides development using computational approach^10^.

Here, we report the first draft assembly of *A. rolfsii* genome and its analysis revealing genes involved in fungicide resistance, virulence and pathogenicity along with putative effector and lethal genes. Our analysis of fungal proteome resulted in the prediction of various secretory proteins involved in carbohydrate metabolism mediating the HP interaction. Evolutionary insight has been elucidated by comparative study of this genome with other ascomycotina fungi. This work would be useful for understanding HP interactions and designing of the strategies for controlling the *A. rolfsii* infection.

## Results and Discussion

### Genome sequencing and assembly

**Figure 1:**
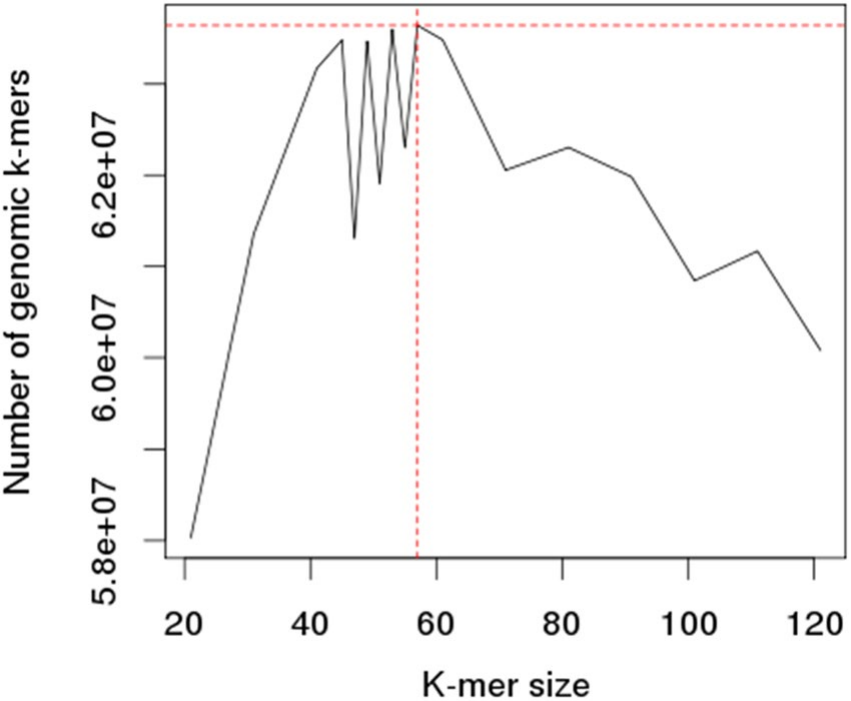
Estimation of genome size and *k*-mer value using *kmergenie* software.

Genome sequencing was performed using Ion torrent PGM and 3.5 Gb of sequence data was generated with average read length 303 bp. High quality reads obtained after trimming of raw reads were subjected for *de novo* genome assembly using MIRA v4.0.2 followed by CAP3 (Table 1). The estimated genome size of ~63.64 Mb was obtained by *k-*mergenie software with optimal *k*-mer size of 57 (Figure 1). An assembly size of 75.37 Mb was obtained using MIRA assembler having 15281 contigs with N50 value of 26586 bp and largest contig length being 249590 bp. Further this assembly was improved using CAP3 software. Finally, we obtained an assembly of size 73.18 MB having 8919 contigs with N50 value of 32103 bp. This improvement of assembly was in magnitude of 41% (reduction in contigs number from 15281 to 8919) and increase in average contig length by ~66% (Table 1). Fungal genome size observed in the study was found to be 73.18 Mb. The potential reasons for difference in estimated and assembled genome size could be: amount of repetitiveness, heterozygosity, read error rate, type of chemistry used, coverage, compromise between contig length and number of errors in assembly^11^. In ascomycotina fungi genome size range varies from 15 (*Babjeviella inositovora*) to 152 (*Zopfia rhizophila*) Mb^12^. Our findings are similar to other reported ascomycotina genomes like *Ascobolus immersus* (59 Mb) and *Terfezia boudieri* (63 Mb)^12^.

**Table 1:** Results obtained after CAP3 assembly.

	Sequences >1 bp	Sequences >500 bp	Sequences >1 kb	Sequences >5 kb	Sequences >10 kb
Total contigs	8,919	7,733	5,222	2,692	1,833
Average contigs length	8,204	9,402	13,586	24,108	32,064
N50 (bp)	32,103	32,380	33,175	36,179	39,338
L50	670	663	636	549	468
Assembly length(bp)	73,180,279	72,709,682	70,949,542	64,900,867	58,773,395

**Figure 2:**
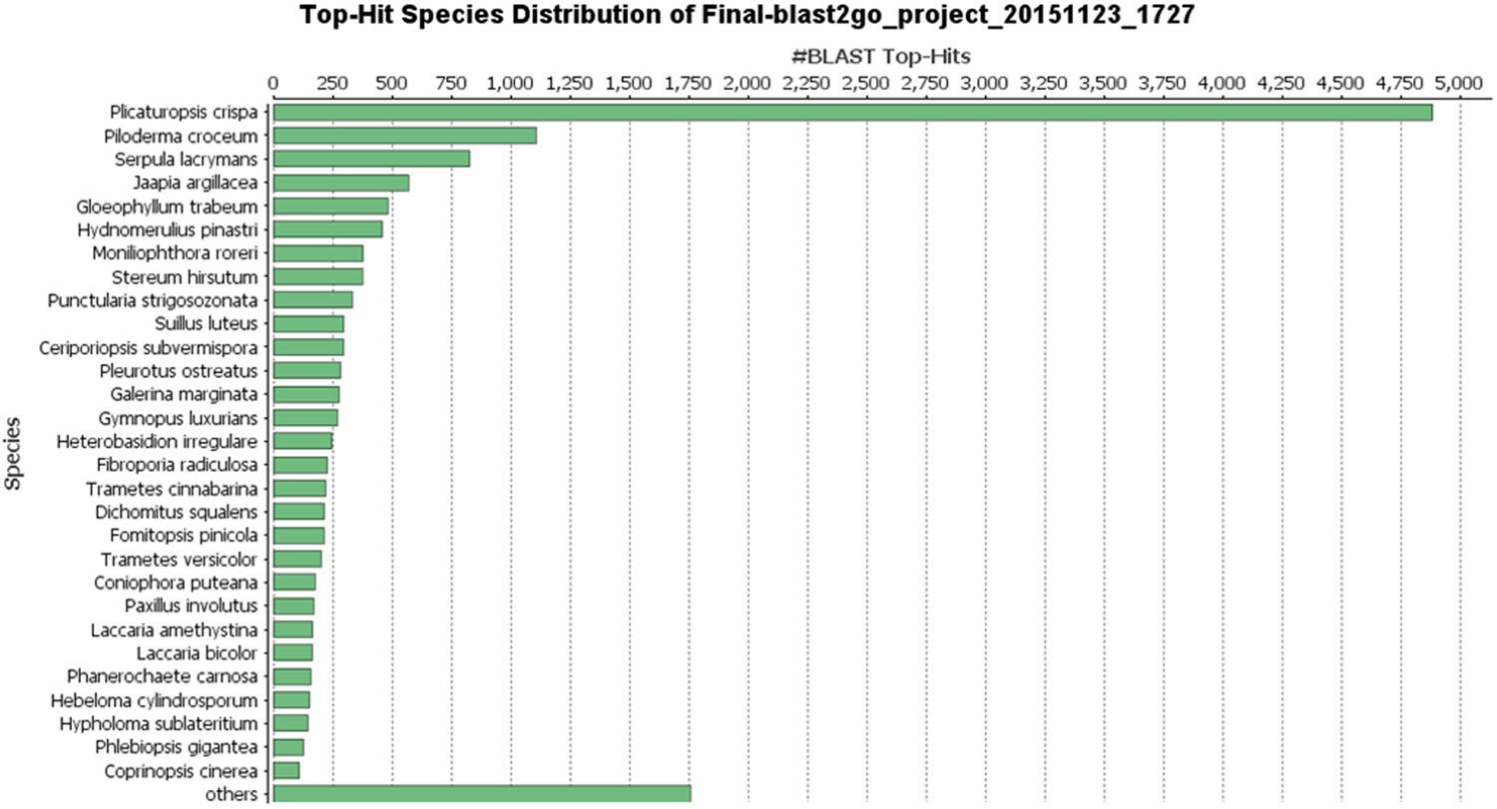
Distribution of blast hits among the different fungal species.

Genemark-ES suite used for gene prediction resulted in a total of 16830 genes. We used 248 core eukaryotic genes (CEGs) using CEGMA pipeline to access the completeness of the genome assembly which revealed hits for 198 CEGs (~80%) with complete match and 229 (~92%) with partial match. Blast2GO Pro version 4.0.7 was used for functional characterization of genes using blastx tool resulted 15292 genes (90.8%) with Blast hits of which 10712 genes (63%) and 5623 genes (33%) were mapped and annotated. Average number of genes in fungal genome are in range of 11129 which varies from 4657 to 27529^12^. The predicted number of genes in our study (16830) is very similar to other ascomycotina like *Anthostoma avocetta* genome (56.23 Mb) having 15755 genes^12^. We observed that ~99% blast hits were obtained from fungal species among which agaricomycetes was in top species and rest (0.96%) were from non-fungal. The maximum number of hits were from *Plicaturopsis crispa* (4884 hits) followed by *Piloderma croceum*, *Serpula lacrymans* and *Jaapia argillacea* (Figure 2).

**Figure 3:**
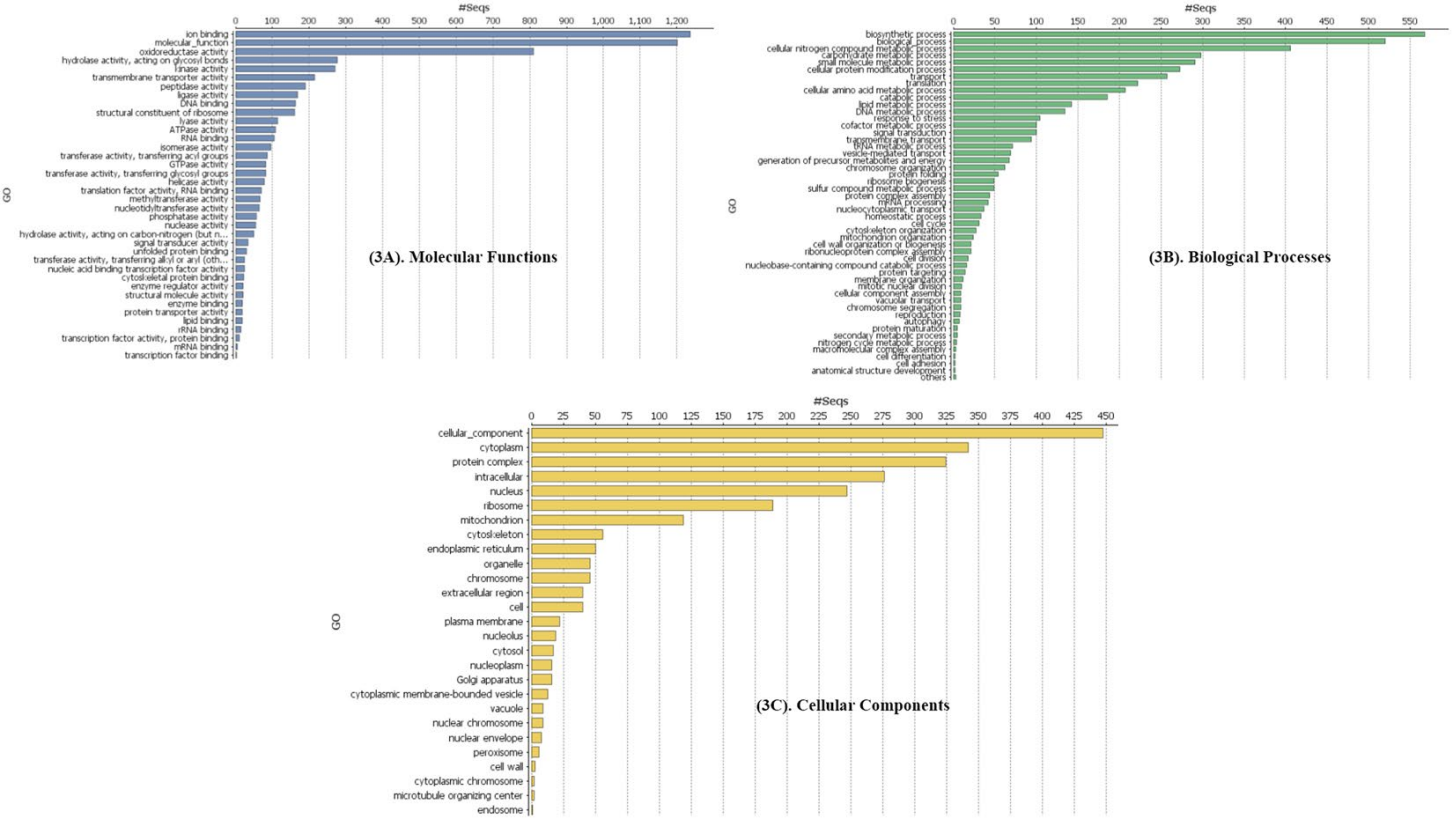
(**A**) GO-term for Molecular function. (**B**) GO-term for Cellular component. (**C**) GO-term for Biological function.

GO level annotation assigned the genes into three groups, *viz*., molecular function, cellular component and biological process. Based on molecular function, 3507 and 261 genes were annotated for catalytic and transporter activities, respectively (Figure 3A). Similarly, biological process classified 343 genes involved in regulation and 237 and 129 genes in stimulus and signaling, respectively (Figure 3B). However, cellular location assigned 795 genes that belonged to organelle and 736 genes were from the membrane (Figure 3C). Fungi are the rich source of secondary metabolites performing many biological functions. These metabolites are directly or indirectly affecting the growth of fungus species and mediation of pathogenicity. Our search for the gene involved in secondary metabolite biosynthesis resulted in the identification of two gene clusters, *viz*., terpene cyclases (TCs) and non-ribosomal peptides synthases (NRPSs) (Supplementary Figure S1)^13^. Both ascomycota and basidiomycota are known to produce various terpenoid compounds as secondary metabolites^14^. Fungal terpenoids play role as toxicant^15^ and also acts for defensive purpose^16^ in pathogenesis. NRPS genes play an important role in positive and negative control of transcription and post-translational events. NRPS gene has diverse gene family coding different peptides as secondary metabolite, such peptides has wide industrial applications like pigments, antibiotics, cytostatics, immunosuppressants, anticancer agents^17, 18^ and in crop protection^19^.

### Protein domain analysis

Pfam domain search based on Pfam database resulted in the identification of 3308 domains distributed in 11624 (69.06%) genes. Maximum number of domains were from WD40 family (1250), followed by CYP450 (283), Pkinase (255), MFS_1 (222), and ABC transporter (106). All of these were known to be involved in HP interaction. The role of these conserved gene families are well reported for example, WD40 in biotic stress tolerance^20^, fungal ABC transporters in virulence^21^, MAPK in fungal growth^22^, pathogenicity and virulence^23^, mechanical/enzymatic penetration in host tissues^24^.

### Repetitive elements and SSR Marker analysis

**Figure 4:**
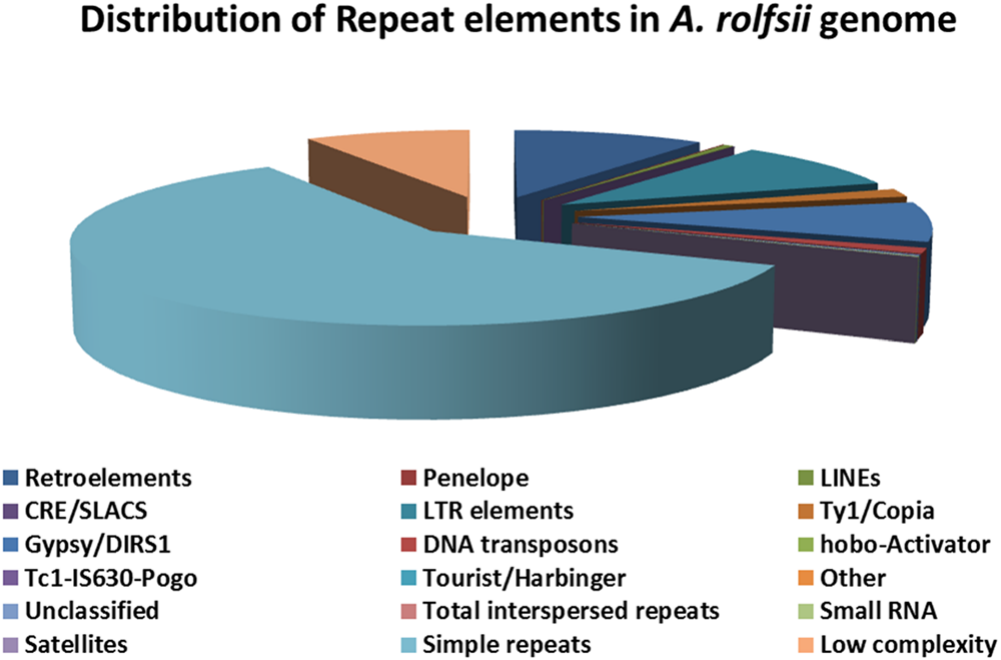
Pie chart of repeats in *A. rolfsii* genome.

Repeats are the most frequently occurring region in case of eukaryotic genome. We used Repeat-masker for identification of repeats in *A. rolfsii* genome that accounts for only 3.73% of the total genome. Fungal genomes contain very less repetitive sequence as compared to other eukaryotes which very rarely exceeds 5% of the genome. The percentage observed in our study is quite consistent of repeat sequences reported in other ascomycete fungi^25^. Highest percentage of repeats were interspersed type (2.69%), small RNA (0.02%), simple repeats (0.85%) and low complexity regions accounting for 0.16% (Figure 4, Supplementary Table S1). LTR, Gypsy, and copia elements were present in higher amount (in 2.51%, 2.18% and 0.33%, respectively) in *A. rolfsii* genome.

Identification of SSRs in *A. rolfsii* genome by whole genome scan revealed a total of 11171 SSRs, including 470 compound SSRs. Mononucleotide SSRs, which were most abundant (9197) represented approximately 82.32% of all SSRs. Among all compound SSRs, 439 were interrupted SSRs (93.4%) and 7 were uninterrupted SSRs. Mono repeats are most abundant in fungal genome^20^. Number of mono repeats varies with fungal species in ascomycotina for example, in *Aspergillus nidulans* (565), *Fusarium graminearum* (371), *Magnaporthe grisea* (3,063), *Neurospora crassa* (2,505), *Saccharomyces cerevisiae* (2,071) and *Schizosaccharomyces pombe* (2,069). In our study, SSR density was found to be 152 repeat length per Mb which is in the reported range of other ascomycotina like *Aspergillus nidulans* (80), *Fusarium graminearum* (80), *Magnaporthe grisea* (307), *Neurospora crassa* (377), *Saccharomyces cerevisiae* (255) and *Schizosaccharomyces pombe* (247)^26^.

GC content of *A. rolfsii* genome was found to be 46.16% which is similar to wood degrading fungus *Phanerochaete chrysosporium* (57%)^26, 27^. The GC and AT regions data can be used in studies related to fungal genome evolution and defense against transposon propagation which operated in AT rich region by repeat-induced point mutation (RIP). Such mechanism has also role in diversifying selection and mediation of host microbe disease interaction^28^. Our reported >11000 SSRs can be used in population structure and diversity analysis which is pivotal in designing strategies for better management of fungal disease. Such approach has been reported in management of leaf and fruit spot fungal disease of citrus^29^. These SSRs can also be used in fungal species/strain differentiation, rapid diagnostics with better sensitivity and specificity^30^.

### Secretome Analysis

**Figure 5:**
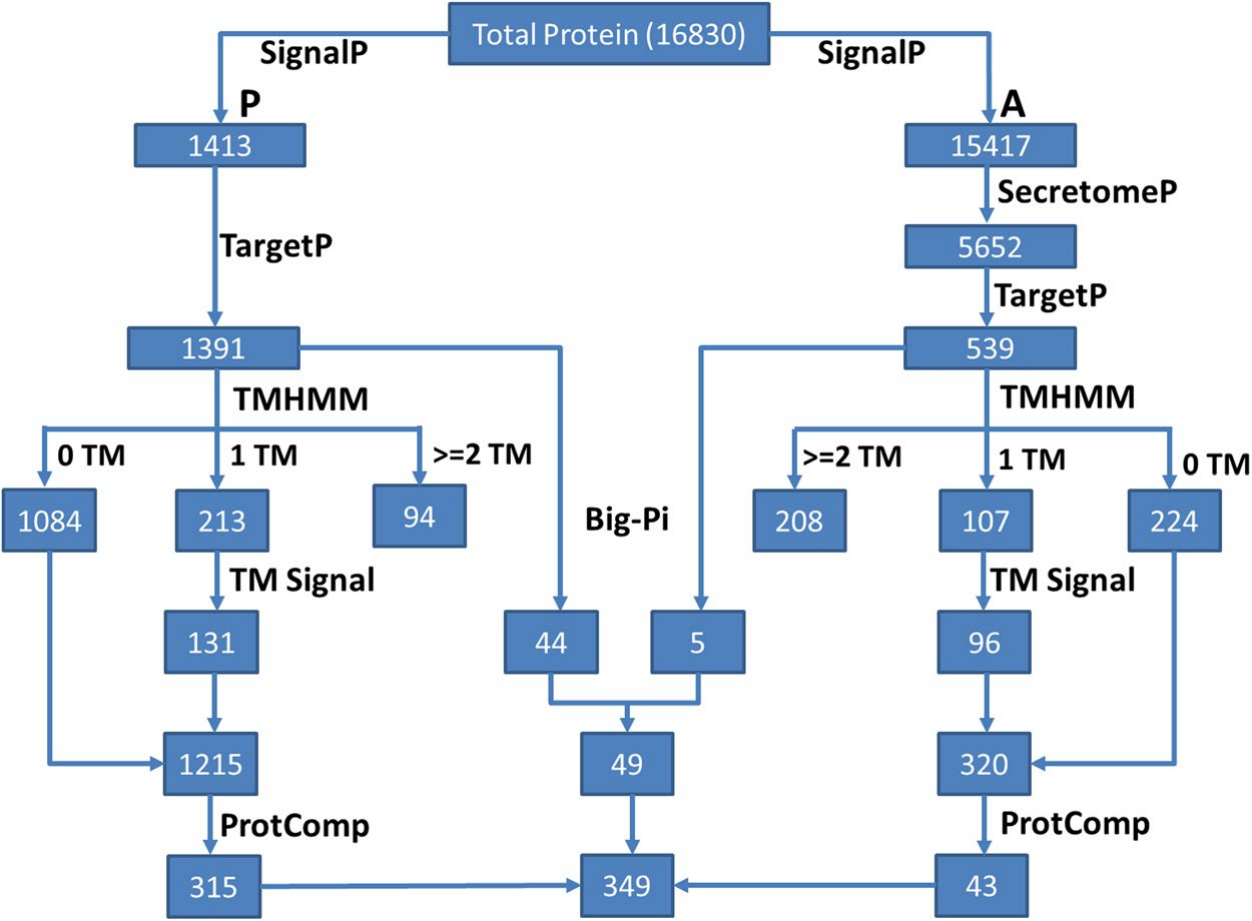
Schematic representation of Secretome Analysis in *A. rolfsii*.

Fungal genome represents the rich source of secretory protein involved in various activities such as signalling, host-pathogen interactions etc. Secretome analysis of *A. rolfsii* genome resulted in the identification of 349 secretory proteins of which 306 were classical and 43 were non-classical secretory proteins (Figure 5). Further analysis of predicted secretome, we observed that 103 genes were assigned for GO-term for cellular location and 197 genes for biological process. Among the top GO-term biological process, cellular process and single cell organism process were highly abundant. Similarly, metabolic process is represented by catalytic and binding activities (Supplementary Figure S2). Under the cellular component, gene for membrane, cell, and cell part were found to be highly abundant. The enzyme annotation revealed four classes of enzymes, *viz*., oxidoreductases, transferases, hydrolases and isomerases. These enzymatic activities represent the high metabolic rate particularly involved in metabolism of host cell wall, an important phenomenon required for fungal invasion into host tissues (Supplementary Figure S3). All such enzymes along with virulence factors or toxins have been reported to degrade the plant cell wall in necrotrophic phase of fungal attack^31^. Pathogen hijacks this process compelling plant to alter cell wall making it more digestible^32^.

### Pathogenicity related genes

**Figure 6:**
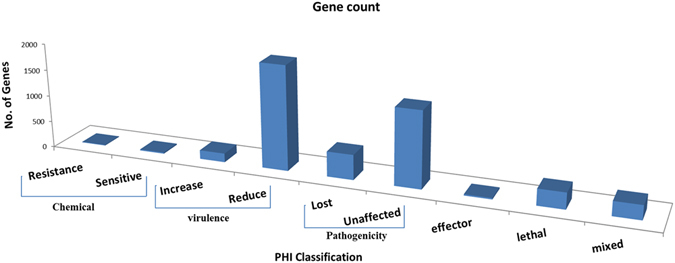
Distribution of *A. rolfsii* pathogenicity related genes in different classes.

Complete proteome of *A. rolfsii* was aligned to PHI database to reveal the pathogenicity related proteins. We observed a total of 4699 (27.99%) PHI genes were classified into different classes such as chemical susceptibility, virulence, pathogenicity, effector, lethal and mixed. As shown in Figure 6, we observed 32 genes associated with chemical susceptibility having 11 resistant and 21 sensitive genes. Furthermore, 309 genes belonged to lethal and 167 to increased virulence class. Remaining three classes *viz*., pathogenicity, effector and mixed were having 1919, 25 and 271 genes, respectively.

Pathogenicity related genes identified in this study have high relevance in future fungicide designing^33^. Mutation in these PR gene actually medicates the sensitivity and resistance of the fungal strain against fungicide^34^. These genes can be targeted for rapid and precise monitoring of fungicide resistance in the field^35^. Our enlisted lethal and virulence genes can be used in future research of fungal disease management, especially by designing of new generation genomic based fungicide^36^. Some of the virulence genes coding for extracellular proteins associated with carbohydrate metabolism have been reported to be involved in horizontal gene transfer (HGT). Such HGT leads to sudden outbreak with very high virulence^37^. The number of lethal genes in our study was found to be 309. In other ascomycotina, for example in *S. cerevisiae*, it was found to have 900 lethal genes^38^.

**Figure 7:**
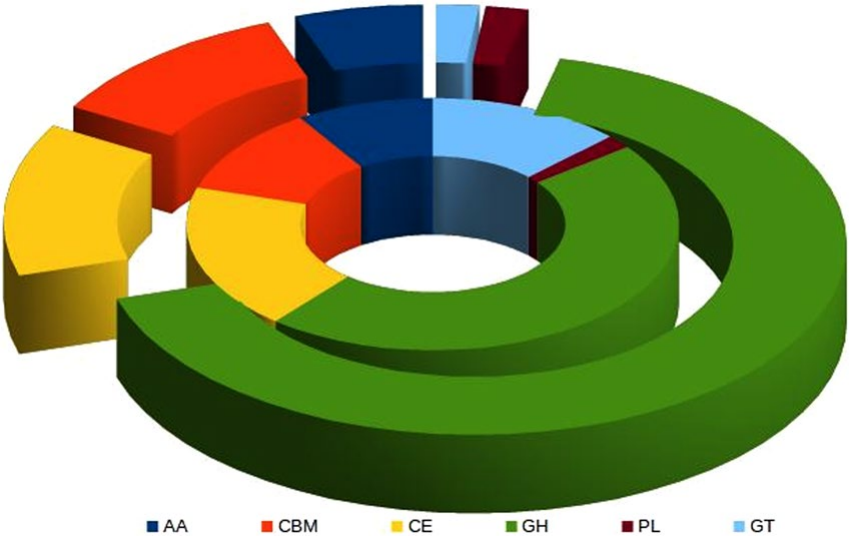
Depicted the distribution of Cazy families into *A.rolfsii* secretome (inner circle) and whole proteome (outer circle). GH: Glycoside Hydrolases, GT: Glycosyl Transferases, PL: Polysaccharide Lyases, CE: Carbohydrate Esterases, CBM: Carbohydrate-Binding Modules, and AA: Auxillary Activities.

CAZymes represents the important component of fungal genome involved in HP interactions. Pathogenic fungus mediates the infection by its action on plant cell wall using the cell wall degrading enzymes. In this work, we identified a sum of 1085 CAZYmes including 54 from the secretory proteins (Supplementary Table S2). As shown Figure 7, maximum number of enzymes were from glycoside hydrolases (GHs), followed by carbohydrate esterases (CEs), carbohydrate-binding modules (CBMs), auxillary activities (AAs), glycosyl transferases (GTs), and polysaccharide lyases (PLs).

Fungal genome often shows very high diversity in CAZymes profile due to their vast diversity in nutritional strategy and host specificity^33^. Large number of CAZymes are produced by fungi for degradation of plant polysaccharide in order to facilitate infection along with nutritional gain^33^. Our study revealed very high number (1085) of CAZymes which was obviously expected as *A. rolfsii* being a necrotrophic fungus. Necrotrophic fungi are reported to have more CAZymes than biotrophic and saprophytic fungi^33^. High abundance of pectinases are also reported in fungal pathogen of dicot like groundnut, with respect to monocot plant. This is due to high abundance of pectin in dicot. Previous phylogenetic studies based on more than 100 fungal species covering similar Basidiomycota along with Ascomycota, Chytridiomycota, and Zygomycota divisions has revealed that these CAZymes have complex history of lineage-specific expansions and attritions^39^.

### Phylogenetic and Orthologous gene family Analysis

**Figure 8:**
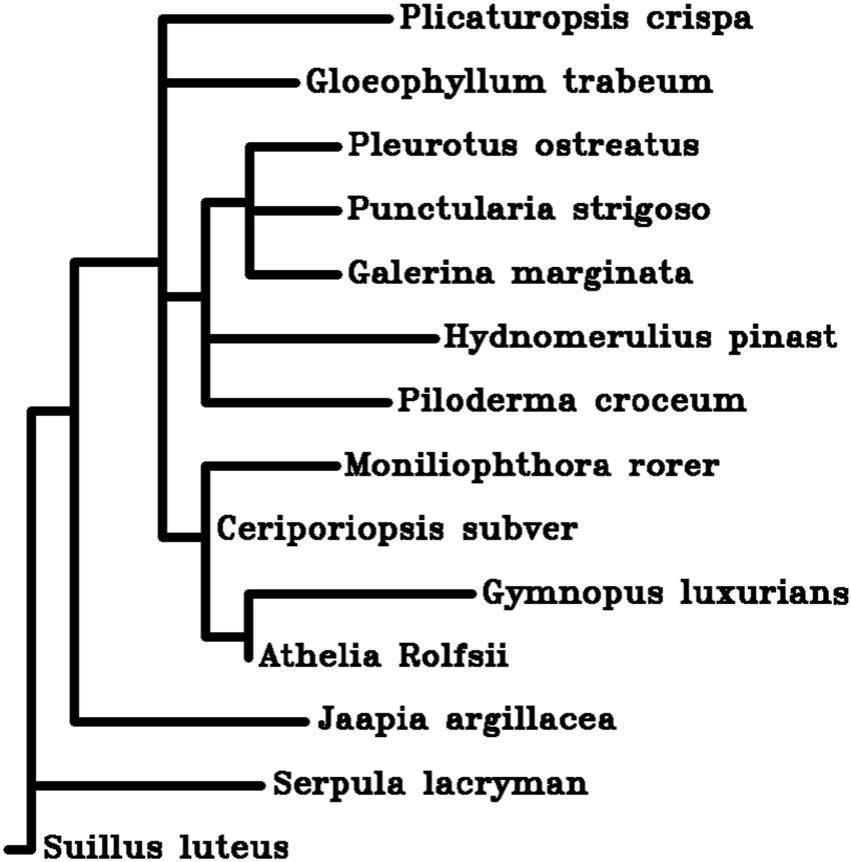
Phylogenetic tree showing the phylogenetic relationship amongst different fungal genomes belonging to class Agaricomycetes.

Whole genome sequences of *A.rolfsii* along with 13 other fungal genomes from agaricomycetes class were used for phylogenetic analysis. As shown in Figure 8, *A.rolfsii* genome appeared to be closely related to *Gymnopus luxuricans* genome. Earlier, ascomycotina fungal species phylogenetic tree has been reported to cover evolutionary history of nutrition^40^. It is observed that in our constructed phylogenetic tree (Figure 8), only one species, *Suillus luteus* is non-saprophytic as it is ectomycorrhiza which is symbiotic^41^.

**Figure 9:**
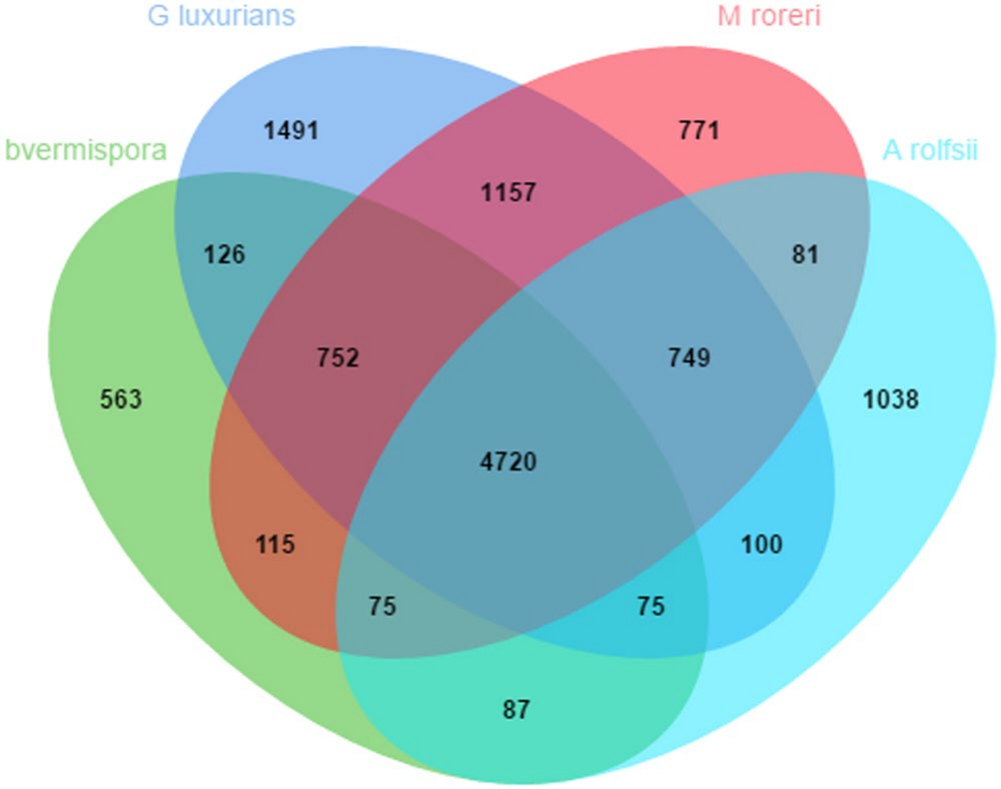
Venn diagram of orthologous gene family among the four fungal species.

Orthologous gene families constitute a clade having *Athelia rolfsii, Gymnopus luxuricans, Ceriporiopsis subvermispora* and *Monoliophthora roreri*. This comparative analysis predicted 4720 core orthologous groups (COGs) (Figure 9). Go enrichment analysis of the COGs identified tri-carboxylic acid (TCA) cycle genes significant at < 0.05 P-value. Among the COGs with significant hits, CYP-452 89A2 is the largest cluster followed by the Zinc finger CCCH domain-containing protein 62, UDP-glycosyltransferase 76E11, and probable voltage-gated potassium channel subunit beta.

The complete proteome of *A. rolfsii* comprises of 16830 genes were clustered into 6925 clustered of which 5887 were shared with other fungal genomes (Figure 9). Furthermore, Go-term enrichment analysis on 1038 unique clusters of *A. rolfsii* resulted in the cell surface (cellular component) and phosphatidylcholine 1-acylhydrolase activity (molecular function) as significant at P-value < 0.05 which plays role in fungal pathogenesis and virulence^42^.

Fungal genome is reported to have extremely variable (>300) Cytochrome P450 proteins (CYPs) genes due to diverse metabolism in their respective ecological niches as an adaptive response. Orthologous studies of CYP genes has high relevance in fungal systematics and classification^35^. Our analysis revealed largest hit with CYP-452 89A2. Agaricomycotina subdivision has been reported to have most dramatic expansion which might be due to gene duplication, adaptive divergence and horizontal gene transfer events^43^. CYP genes has potential to be evaluated for fungal diagnostics as reported in other fungi like *Candida spp* ^44^. Conserved orthologous gene family UDP-glycosyltransferase 76E11 are reported to be associated with glyco-diversification of bioactive natural products having relevance in drug discovery. Similarly, the conserved gene family, probable voltage-gated potassium channel subunit beta is relevant in future studies on fungicide sensitivity^45^.

## Conclusion

This is the first report of whole genome sequence based draft assembly of *Athelia rolfsii*, causing important fungal disease, stem rot which affects groundnut productivity in many countries. Knowledge discovery from this studies will not only provide new insight in understanding the pathogenicity and virulence but will also lead to new dimension in development of disease combating strategies. Such more studies are required having transcriptomic and proteomic approach for genomic solution in the productivity management endeavour of its oil producing host crop.

## Methodology

### DNA Isolation

*Athelia rolfsii* was isolated from an infected groundnut plant variety (GG-20) at Junagadh Agricultural University, Gujarat, India (21.51° N, 70.45° E). Potato Dextrose Agar (PDA) medium was used for growth and maintenance of isolates. These cultures were stored at 4 °C for long-term storage. *Athelia rolfsii* strains were grown in PDA broth medium for 6-days at 200 rpm at 28 °C. Mycelia were filtered and genomic DNA was extracted using Hipur ATM Fungal DNA purification kit (Himedia Cat. No. MB543) as per manufacturer’s protocol.

### DNA library construction and Ion torrent PGM sequencing

Library construction was conducted as per the Ion plus fragment library kit (Invitrogen Cat. No 4471252) for whole genome libraries. Total genomic DNA input was 100 ng which was fragmented using Ion shear^TM^ enzyme mix II enzyme with an average of 400 bp DNA fragment sizes. The fragmented genomic library was cleaned using Agencourt Ampure XP Reagent (Beckman Coulter). The fragmented DNA was quantified using the Qubit DSDNA HsAssay Kit with the Qubit Fluorometer (Invitrogen). Purified DNA fragments were ligated with adapters specific to cleavage site of endonuclease enzyme, followed by the size selection of genomic library using E-gel 2% in order to get fragment size of 350–400 bp. The library was amplified using 10 cycles of PCR for enrichment of adapter ligated fragments and purified with Agencourt Ampure XP Reagent (Beckman Coulter). Template preparation for sequencing was conducted according to the OneTouch Ion™ Template Kit (Life Technologies). The selected PCR products were again used for emulsion PCR, followed by positive bead recovery. Ion Torrent sequencing was conducted using the Ion PGM^TM^ 400 Sequencing Kit (Life Technologies) on an Ion Torrent Personal Genome Machine (PGM^TM^, Life Technologies) using a 318V^2^-chip (Ion 318^TM^ chip, Life Technologies).

### Data pre-processing and genome assembly

Reads were processed using Fastx toolkit v0.013 (http://hannonlab.cshl.edu/fastx_toolkit/) and bases having phred score (Q) less than 20 were trimmed. High quality processed reads were used for estimation of genome size using kmergenie v1.69972 tool^46^. *De novo* assembly were performed using MIRA v4.0.2^47^ assembler followed by CAP3 software^48^ to obtain final draft genome assembly of *A. rolfsii*.

### Gene prediction and annotation

RepeatMasker (http://www.repeatmasker.org/) was used to mask the repeats in contigs by selecting fungi as species and rmblastn as the search engine with slow search option. To search the genes in draft genome, Genemark-ES suite v4.21^49^ was used with following parameters –ES (self-training to predict genes); –fungus; –min-contig size: 500; –max-intron size: 3000; –min_gene_prediction: 120. Cufflinks was used for extracting coding and exon sequence in fasta format using –J option^50^. Presence of full length genes from the assembly was confirmed by using ORFfinder tool^51^. CEGMA v2.5^52^ was used to assess the completeness of the genome assembly and functional annotation of identified genes was done using Blast2GO Pro Ver. 4.0.7 software^53^. To access the domains in the genome of *Athelia rolfsii*, the assembly was analysed against Pfam^54^ database at e-value cutoff 1e-05. The assembled genome was further used for searching genes involved in secondary metabolite biosynthesis using antiSMASH webserver^55^.

### Secretory protein prediction and its analysis

All predicted proteins of *Athelia rolfsii* was used as input for identification and analysis of secretory proteins. SignalP v4.1^56^ was used for the prediction of the signal peptide and those lacking the signal peptide were analyzed by SecretomeP v1.027^57^. Further, the output obtained from both tools were analysed using TargetP v1.1^58^. Subsequently, transmembrane domains and GPI (glycosylphosphatidyl inositol)-anchor were predicted using TMHMM v2.0^59^ and big-PI FungalPredictor tool^60^, respectively.

Proteins having no transmembrane and one transmembrane domain within N-terminal Signal peptides were enlisted. Further, the proteins obtained from both classical and non-classical pipeline were subjected to sub-cellular localization prediction using ProtComp v9.0 based on LocDB and PotLocDB databases (http://www.softberry.com). Finally, GPI-anchor proteins were filtered out from the final set and rest of the sequences were the designated as secretome.

### Identification of the pathogenicity related genes

PHI-base (a database of Host-Pathogen gene interactions)^61^ was used to search the pathogenicity related gene in query sequence using Blastp. We also used dbCAN webserver^62^ with default parameter to search the genes for their function as carbohydrate active enzyme (CAZY). This search was based on CAZY database which classified the hit into different families according to the type of reaction being catalyzed.

### Phylogenetic relationships

Whole genome sequence of 13 fungal species belonging to same class, Agaricomycetes were downloaded from NCBI (http://ncbi.nlm.nih.gov/). Phylogenetic tree was constructed using RealPhy software^63^ and visualized using the Drawgram module of Phylip tool v3.695^64^. This tool takes number of genomic sequences as input, uses bowtie2 for read mapping and constructs a phylip file containing the input genomic sequences^65^.

### Search for Orthologous gene family

The complete proteome of the three fungal species i.e. *A. rolfsii, G. luxuricans, C. subvermispora, M. roreri* were downloaded from NCBI database. Orthovenn server^66^ was used for identification of shared and unique orthologous gene families clusters in the above mentioned fungal genomes.

### Data Deposition

The whole-genome sequence and annotation of *Athelia rolfsii* isolate MR10 have been deposited at NCBI (https://www.ncbi.nlm.nih.gov/) with accession JZWR00000000; BioSample SAMN03388249.

## Electronic supplementary material

Supplementary Tables and Supplementary Figures
