# Supplementary material for: Draft whole genome sequence of groundnut stem rot fungus Athelia rolfsii revealing genetic architect of its pathogenicity and virulence
Source: Sci Rep. 2017 Jul 13;7:5299. doi: 10.1038/s41598-017-05478-8 (PMC5509663; doi:10.1038/s41598-017-05478-8)
Supplement: Supplementary file 1 — Supplementary Tables and Supplementary Figures [file 41598_2017_5478_MOESM1_ESM.doc]

**Draft whole genome sequence of groundnut stem rot fungus *Athelia rolfsii* revealing genetic architect of its pathogenicity and virulence**

MA Iquebal^1#^, Rukam S Tomar^2#^, MV Parakhia^2^, Deepak Singla^1^, Sarika Jaiswal^1^, VM Rathod^2^, SM Padhiyar^2^, Neeraj Kumar^1^, Anil Rai^1^, Dinesh Kumar^1,*^

**Authors**

^1^Centre for Agricultural Bioinformatics, ICAR-Indian Agricultural Statistics Research Institute, Library Avenue, PUSA, New Delhi-110012, INDIA

^2^Junagadh Agricultural University, Junagadh 362 001, Gujarat, INDIA

**Supplementary Information**

**Supplementary Table S1: Distribution of repeat elements in *A. rolfsii* genome.**

**Supplementary Table S2: Number of proteins from different CAZY families**

**Supplementary Table S1: Distribution of repeat elements in *A. rolfsii* genome.**

| **Repeat Types** | **No. of elements** | **Percentage of Genome (%)** |
| --- | --- | --- |
| Retroelements | 2841 | 2.54 |
| SINEs | 0 | 0.0 |
| Penelope | 14 | 0.02 |
| LINEs | 145 | 0.03 |
| CRE/SLACS | 1 | 0.0 |
| L2/CR1/Rex | 0 | 0.00 |
| R1/LOA/Jockey | 0 | 0.00 |
| R2/R4/NeSL | 0 | 0.00 |
| RTE/Bov-B | 0 | 0.00 |
| L1/CIN4 | 0 | 0.00 |
| LTR elements | 2696 | 2.51 |
| BEL/Pao | 0 | 0.00 |
| Ty1/Copia | 469 | 0.33 |
| Gypsy/DIRS1 | 2225 | 2.18 |
| Retroviral | 0 | 0.00 |
| DNA transposons | 305 | 0.16 |
| hobo-Activator | 4 | 0.00 |
| Tc1-IS630-Pogo | 43 | 0.01 |
| En-Spm | 0 | 0.00 |
| MuDR-IS905 | 0 | 0.00 |
| PiggyBac | 0 | 0.00 |
| Tourist/Harbinger | 17 | 0.00 |
| Other (Mirage, P-element, Transib) | 1 | 0.00 |
| Rolling-circles | 0 | 0.00 |
| Unclassified | 15 | 0.00 |
| Total interspersed repeats |  | 2.69 |
| Small RNA | 39 | 0.02 |
| Satellites | 3 | 0.00 |
| Simple repeats | 16797 | 0.85 |
| Low complexity | 2444 | 0.16 |

**Supplementary Table S2: Number of proteins from different CAZY families**

| **CAZY Family** | **Secretary Protein** | **CAZymes** |
| --- | --- | --- |
| AA | 3 | 98 |
| CBM | 6 | 122 |
| CE | 7 | 203 |
| GH | 36 | 505 |
| PL | 1 | 18 |
| GT | 1 | 139 |
| Total | 54 | 1085 |

**Supplementary Information**

**Supplementary Figure S1: Metabolite gene cluster in *A. rolfsii* genome of two contigs 13 and 385**

**Supplementary Figure S2: Top Go-term annotation of *A. rolfsii* secretome**

**Supplementary Figure S3: Domain characteristics of *A. rolfsii* secretome**

| 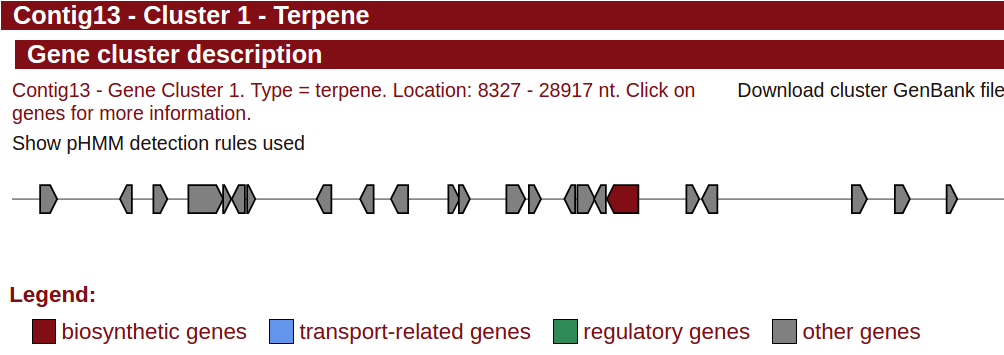 |
| --- |
|  |
| 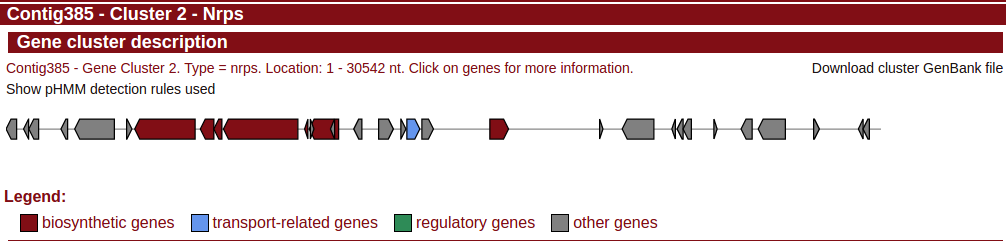 |

**Supplementary Figure S1: Metabolite gene cluster in *A. rolfsii* genome of two contigs 13 and 385**

| **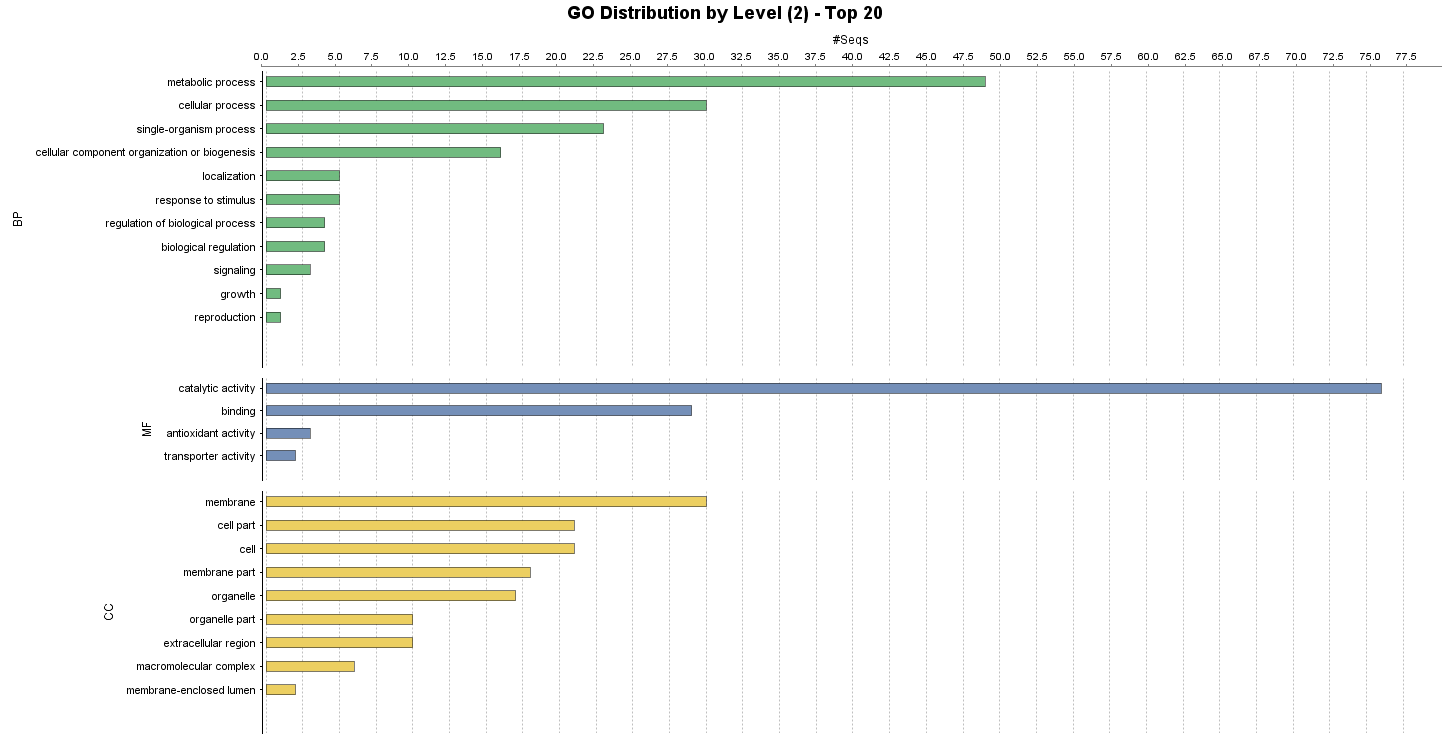** |
| --- |
| **Supplementary Figure S2: Top Go-term annotation of *A. rolfsii* secretome** |
| **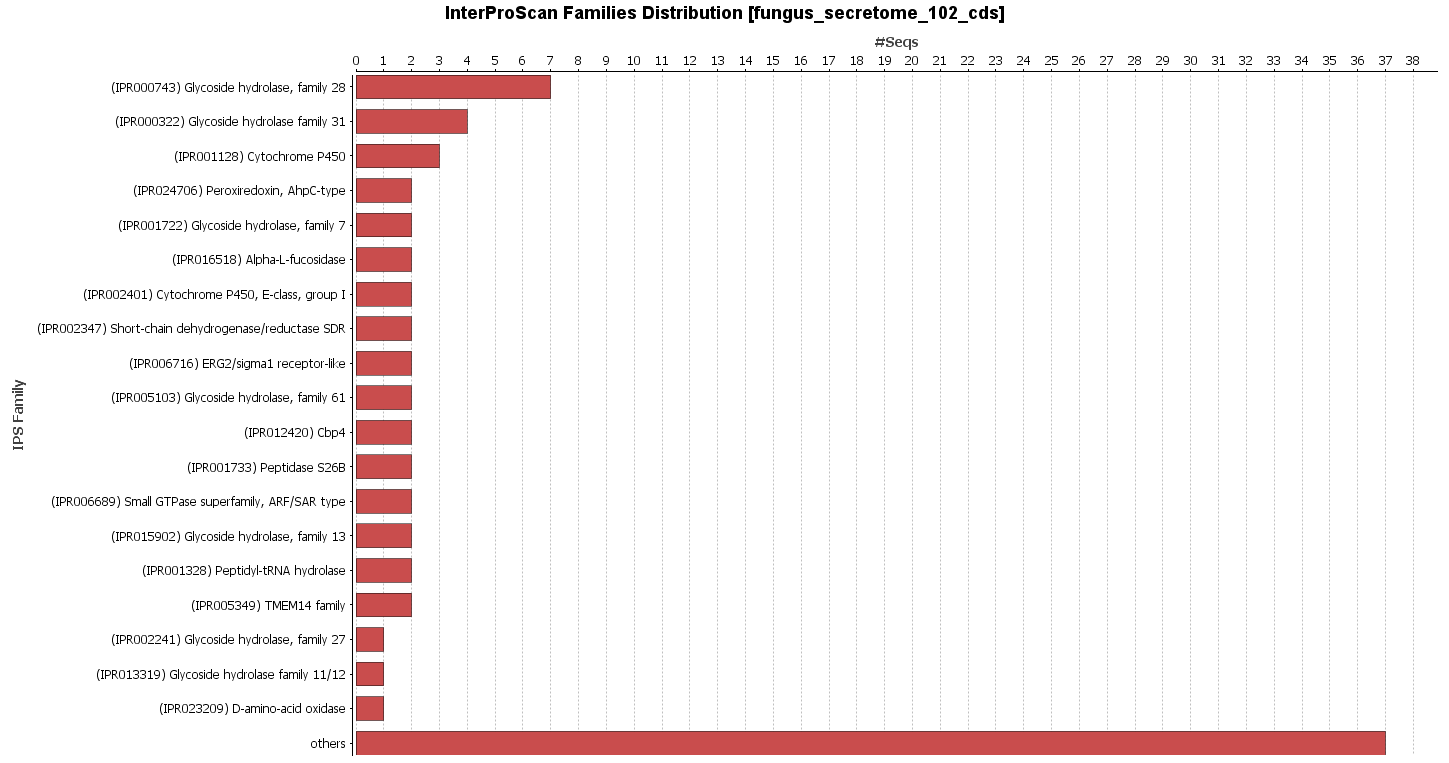** |
| **Supplementary Figure S3: Domain characteristics of *A. rolfsii* secretome** |
